# Supplementary material for: Health-related quality of life among women aging with and without HIV in Peru
Source: PLoS One. 2022 Jun 9;17(6):e0269000. doi: 10.1371/journal.pone.0269000 (PMC9182248; doi:10.1371/journal.pone.0269000)
Supplement: Attachment — Submitted filename: Response to Reviewers_12.22.21.docx [file pone.0269000.s002.docx]

December 22, 2021

Sebastian Shepherd

Associate Editor

PLOS ONE

We appreciate your comments and suggestions. Please see below the responses for both journal and reviewers’ comments.

**Journal Requirements**

1. Please ensure that your manuscript meets PLOS ONE's style requirements, including those for file naming. The PLOS ONE style templates can be found at 
https://journals.plos.org/plosone/s/file?id=wjVg/PLOSOne_formatting_sample_main_body.pdf and 
https://journals.plos.org/plosone/s/file?id=ba62/PLOSOne_formatting_sample_title_authors_affiliations.pdf

**Response: We appreciate you pointing this out. We have double-checked that the manuscript meets PLOS ONE’s style requirements.**

2. Please review your reference list to ensure that it is complete and correct. If you have cited papers that have been retracted, please include the rationale for doing so in the manuscript text, or remove these references and replace them with relevant current references. Any changes to the reference list should be mentioned in the rebuttal letter that accompanies your revised manuscript. If you need to cite a retracted article, indicate the article’s retracted status in the References list and also include a citation and full reference for the retraction notice.

**Response: We appreciate you pointing this out. We have reviewed and our reference list is complete and correct.**

3. We note that the grant information you provided in the ‘Funding Information’ and ‘Financial Disclosure’ sections do not match.

When you resubmit, please ensure that you provide the correct grant numbers for the awards you received for your study in the ‘Funding Information’ section.

**Response: Thank you so much for letting us know about this. We have ensured that our grant number and funding information is correct. Our only funding source was the Fogarty International Center grant D43TW010540.**

4. We note that you have stated that you will provide repository information for your data at acceptance. Should your manuscript be accepted for publication, we will hold it until you provide the relevant accession numbers or DOIs necessary to access your data. If you wish to make changes to your Data Availability statement, please describe these changes in your cover letter and we will update your Data Availability statement to reflect the information you provide.

**Response: We have uploaded our project data information in the Open Science Framework repository. It can be accessible through this link: https://osf.io/a95vr/?view_only=e51537b490e04798902de7975e742c20.**

5. Please note that in order to use the direct billing option the corresponding author must be affiliated with the chosen institute. Please either amend your manuscript to change the affiliation or corresponding author, or email us at [plosone@plos.org](mailto:plosone@plos.org) with a request to remove this option.

**Response: We appreciate you pointing this out. The corresponding author is Dr. Evelyn Hsieh affiliated with Yale School of Medicine.**

**Comments to the Author**

1. Is the manuscript technically sound, and do the data support the conclusions?

The manuscript must describe a technically sound piece of scientific research with data that supports the conclusions. Experiments must have been conducted rigorously, with appropriate controls, replication, and sample sizes. The conclusions must be drawn appropriately based on the data presented.

Reviewer #1: Yes

Reviewer #2: Yes

Reviewer #3: Yes

**Response: We appreciate your comments on this.**

2. Has the statistical analysis been performed appropriately and rigorously?

Reviewer #1: Yes

Reviewer #2: Yes

Reviewer #3: Yes

**Response: We appreciate your comments on this.**

3. Have the authors made all data underlying the findings in their manuscript fully available?

The [PLOS Data policy](https://nam12.safelinks.protection.outlook.com/?url=http%3A%2F%2Fwww.plosone.org%2Fstatic%2Fpolicies.action%23sharing&data=04%7C01%7Cdiego.cabrera%40yale.edu%7C81bc54c00ebe4bba7df608d988fc9ace%7Cdd8cbebb21394df8b4114e3e87abeb5c%7C0%7C0%7C637691440886062626%7CUnknown%7CTWFpbGZsb3d8eyJWIjoiMC4wLjAwMDAiLCJQIjoiV2luMzIiLCJBTiI6Ik1haWwiLCJXVCI6Mn0%3D%7C1000&sdata=hpgodpzlWiNUXDWOaDzcxi%2FMLedAWZV4cMpIL%2Fly6uk%3D&reserved=0) requires authors to make all data underlying the findings described in their manuscript fully available without restriction, with rare exception (please refer to the Data Availability Statement in the manuscript PDF file). The data should be provided as part of the manuscript or its supporting information, or deposited to a public repository. For example, in addition to summary statistics, the data points behind means, medians and variance measures should be available. If there are restrictions on publicly sharing data—e.g. participant privacy or use of data from a third party—those must be specified.

Reviewer #1: Yes

Reviewer #2: No

Reviewer #3: No

**Response: We appreciate your comments on this. We have uploaded our project data information in the Open Science Framework repository. It can be accessible through this link: https://osf.io/a95vr/?view_only=e51537b490e04798902de7975e742c20.**

4. Is the manuscript presented in an intelligible fashion and written in standard English?

PLOS ONE does not copyedit accepted manuscripts, so the language in submitted articles must be clear, correct, and unambiguous. Any typographical or grammatical errors should be corrected at revision, so please note any specific errors here.

Reviewer #1: Yes

Reviewer #2: Yes

Reviewer #3: Yes

**Response: We appreciate your comments on this.**

5. Review Comments to the Author

Please use the space provided to explain your answers to the questions above. You may also include additional comments for the author, including concerns about dual publication, research ethics, or publication ethics. (Please upload your review as an attachment if it exceeds 20,000 characters)

Reviewer #1: This is a very important paper on physical activity of women living with HIV.

SF-36 PCS and MCS scores transformation to have a mean of 50 and standard deviation of 10 should be better described for readers not familiarized with SF-36. Alcohol and smoking status dichotomous assessment represents a limitation.

**Response to Reviewer #1: We thank the reviewer for these insightful comments. We have expanded on how the component scores were calculated and transformed (lines 175-179, page 7).**

The authors should explore more the association of physical activity; it has affected both physical and mental status in the HIV-infected subset in opposite directions. What hypothesis could be formulated?

**Response to Reviewer #1: Regarding the differing effects of physical activity on both physical and mental status, we have hypothesized that physical activity levels are comprised of not only intentional exercise but also unintentional activities such as excessive manual labor. Physical health may be positively influenced by both intentional and unintentional exercise but mental health may be negatively impacted by the latter one (page 21, lines 456-460).**

In the limitations paragraph the authors states that “findings cannot be extrapolated to represent HIV-infected women outside of Lima”. It should be rephrased. It lacks external validity even for HIV-infected women living in Lima, since it was not a probabilistic sample and was a hospital-based study.

**Response to Reviewer #1: Regarding the mentioned limitation, we have now modified the wording to reflect the concern raised by the reviewer (lines 427-430, page 20)**

Is there any plan to use SF-36 data in further health economic evaluations of technologies to improve physical activity for this population?

**Response to Reviewer #1: We are aiming to share this publication with the National HIV/STI and Hepatitis Program in Peru in order to inform them about our findings and have discussions about potential steps the National Program can take to improve both physical and mental health on this population. We thank the reviewer for this specific suggestion and will take it into consideration.**

Reviewer #2: The manuscript “Health-Related Quality of Life among Women Aging with and without HIV in Peru” compares health related quality of life (measured by the SF-36) among aging women living with and without HIV and predictors of health-related quality of life among these two samples. There are some interesting and surprising findings and I commend authors for exploring these issues among a population that is rarely studied. There were a few areas that, if addressed, would enhance the manuscript:

• Introduction
o It seems like the introduction could be improved by including directional hypotheses. Did authors predict that women living with HIV would have worse health related quality of life than those without HIV? Why did they predict this?
o Additionally, it seems like there are some important contextual factors missing from the examination of health-related quality of life. For example, what would the literature suggest about the impact of illness, stigma, etc. on health in this population? What are important contextual factors specific to Peru? Are there specific policies in Peru? Histories of stigma? Impact of religious stigma? Etc. Even a few sentences might “set up” why this is an important question in this location (other than that it has not been studied before). Authors point to the “unequal status of women” – how is that related to sexual health empowerment or lack thereof in Peru? How does that impact the context in which living with HIV is experienced?

**Response to Reviewer #2: We thank the reviewer for these comments. In terms of the Introduction, the hypothesis statement is included (lines 104-105, page 4) and we have added information (lines 106-107, page 4) regarding the impact of HIV and stigma among women with HIV in the context of Latin American and Peru and how these factors negatively impact their health.**

• Methods
o Smoking history as “ever vs. never” seems less important to me than “current vs. not current.” Within the U.S. people living with HIV are much more likely to smoke cigarettes than the general population. Is this the same for Peru? It would have been nice to know whether individuals are current smokers, and this likely would be much more highly correlated with current health and health related quality of life than “ever” smoking status. This is particularly true because of the age of individuals – many may have smoked when they were younger and have since stopped. Without this information (or even years of smoking) the impact of this health behavior is less meaningful.
o Although the Spanish version of the SF-36 was used, I wonder if authors could comment on the cultural appropriateness of answering these questions within a medical setting in Peru. During pilot testing, did any patients comment on answering these questions? Did they seem comfortable giving a range of responses? I’m wondering if there is a chance that people may have answered in a socially desirable way because of cultural expectations or norms about health vs. illness. While outside the scope of this paper it might be worth commenting on the appropriateness of the use of this measure within the methods and/or acknowledging potential limitations in the discussion.

**Response to Reviewer #2: Regarding the Methods, thank you for your suggestion of measuring the variable for smoking as "current vs. not current", and we will considering this measure for future studies. Regarding the Spanish version of the SF-36, yes we did pilot this version prior to starting recruitment to assess cultural appropriateness and ease of understanding. We received no negative feedback or from the pilot sample, and therefore did not need to modify it. Additionally, this version has previously been validated and used successfully among other Latin American populations, which factored into our decision to utilize this version.**

• Results
o Time since diagnosis and ART duration are presented as mean values with standard deviations. It would have also been nice to see the range of values for the sample and perhaps even the median value.
o HIV-related health values like CD4+ cell count and nadir and current viral load should also present median values. Mean values could be misrepresentative if there are extreme outliers.
o Current viral load mean seems extremely high for a population engaged in care. What could explain viral loads of 21,000+? This relates to my prior comment about presenting median values instead of means as this may be skewed by high outlier values. Typically, log viral load is what is presented in research – is that what these values are? Or have they not been converted to log values yet? Additionally, it would be beneficial to see how many were considered undetectable (different definitions by generally, <200 has been used internationally, <20 log viral load is used in most clinical settings in the U.S.) versus having a detectable viral load. It would make sense to look at the impact of viral load in this way as well.

**Response to Reviewer #2: We thank the reviewer for these suggestions. In terms of the Results, we have updated the median values and interval quartiles in Table 1 (page 10) for current CD4+ T-cell count, ART-naive CD4+ T-cell count, time since HIV diagnosis and ART duration, and ART-naive and current HIV viral load. We have also included the proportion of patients who were virally suppressed, defined as a current viral load of ≤50 copies.**

• Discussion
o There is a statement made about “sociodemographic contributing to health” because mestizo-identified women had worse health than white identified women. However, I think this is better reframed as mestizo-identified women experienced worse self-reported health likely as a result of social forces and experiences. It might be worth describing how race and social status, health, etc. are related in general, within the global South, and/or within Peru.
o In general, there were some surprising findings that would benefit from additional post-hoc explanations and references to literature. For example, current alcohol use was associated with better health among women living with HIV and women without HIV. Why do authors think this is true? One potential explanation that came to mind is that it is possible individuals who drink alcohol are also socializing more, and that social support often promotes better health. Authors should explore this and other potential explanations for some of there findings rather than just leaving them.
o Another surprising finding was that women living with HIV reported fewer role limitations due to physical or emotional health. Why is this? Is it that they are employing perspective taking? Are happy about their health considering their HIV diagnoses? Could it be that they are more highly engaged in caring for their health because of HIV? That they seek medical care more often through an infectious disease clinic? Authors do not need to know the reasons for these surprising findings, but I do believe it would be beneficial to explore potential explanations in the context of plausible conclusions based on their knowledge of the sociocultural context, literature, etc.
o Within the limitations, authors state that their findings cannot be extrapolated or generalized outside of the specific city of Lima. Why is this? Is the sample in Lima not representative of Peru in general?
o In the final paragraph, authors acknowledge potential next steps in research, however, they are missing one somewhat obvious next step which is to understand how women living with HIV in Peru are thriving. This sample seems to report better health than those without HIV and maybe there are mental, social, or lifestyle behaviors that if identified could be leveraged to improve the health of women living with HIV. Rather than a deficit focus, this paper nicely lends itself to a resiliency focus of people living with HIV and this should be emphasized within the conclusion and carried throughout to strengthen the findings.

**Response to Reviewer #2: In terms of the Discussion, we have updated the sociodemographic contributing aspects in terms of ethnicity on page 18 (lines 377-380), describing how race and social status are closely related in Peru. In terms of current alcohol use, although this was associated with higher physical component scores among both HIV-infected and uninfected women in the univariate regression analysis (Table 3, page 13-14), it was no longer statistically significant for the multivariable analysis among HIV-infected women – only for the uninfected women. This could possibly be due to socializing effects promoting better physical health, however, we did not explore those factors in depth in the discussion as our primary focus for this manuscript was on factors impacting the HIV-infected women. In terms of role limitations due to physical and emotional health, we thank the reviewer very much for this important insight, we have added additional content (page 17, lines 345-351) regarding the potential cause of these lower scores among HIV-infected women, possibility because these women are engaged in continuous care and can address these issues with their doctor. Regarding the limitations, although we have worked with a representative sample from a one of the largest HIV clinics in Lima (the capital city of Peru) which provides care to a diverse population, as with any single-center study there is a potential limit to generalizability (as pointed out by Reviewer 1). Therefore we wanted to mention this so that potential readers have this in mind when analyzing this paper. In terms of the final paragraph, while overall physical and mental component scores were not significantly different between the two groups, scores were significantly lower among women with HIV for several individual domains (indicated worse HRQoL). We have clarified this point in the conclusion paragraph. We agree it is valuable to consider how women with HIV in Latin America are thriving as a means of identifying resiliency factors in future studies, so have added this concept as well on page 22 (lines 465-468).**

Reviewer #3: Review Report
Title: Health-Related Quality of Life among Women Aging with and without HIV in Peru
Reviewer: Prof. Sam Ibeneme
Institutional affiliations:
1. University of Nigeria, Enugu Campus, Department of Medical Rehabilitation, Faculty of Health Sciences, Enugu, Nigeria. [sam.ibeneme@unn.edu.ng](mailto:sam.ibeneme@unn.edu.ng).
2. Department of Physiotherapy, Faculty of Health Sciences, School of Therapeutic Studies, University of the Witwatersrand, 7 York Road, Parktown, 2193 Johannesburg, South Africa

Reviewers Report
General comments
Many thanks for inviting me to review the suitability of the article Health-Related Quality of Life among Women Aging with and without HIV in Peru’ for publication. Essentially, this article sought to (1) determine whether the effects of aging on HRQOL vary in aging women with or without HIV infections, and (2) to explore the explanatory variables for the variance in HRQOL in aging women with or without HIV infections using predictive models. Therefore, they compared older adult women with and without HIV infection to know whether the impact of aging on HRQOL is similar in both groups or whether there are differences in both groups which could be accounted for by HIV-related factors. The authors also explored the predictive models that could explain differences in HRQOL between the older adult women with or without HIV infection. The findings seem to demonstrate that HRQOL is more negatively affected among older adult women with HIV than otherwise. The summary score for the physical component of HRQOL in the HIV subset was significantly associated with physical activity, ethnicity, and chronic comorbidities suggesting a role for physical activity, socio-cultural factors and HIV-related/age-related complications in the determination of the physical wellbeing of the aging HIV-infected women. Similarly, the mental component was significantly associated with physical activity, employment, and CD4+ cell count suggesting a role for a physical activity lifestyle, social functioning/economic factors and symptoms of the disease severity in the determination of the emotional wellbeing of the aging HIV-infected individuals. Overall, it was shown that HRQOL may have important clinical application in the early identification of the key drivers of physical and emotional welling in the aging HIV infected individuals. These drivers should be prioritised in the clinical management and policy interventions of HIV disease which may vary across socio-cultural boundaries considering the impact of ethnicity. Thus, I consider this study to be original, useful, timely and relevant.

**Response to Reviewer #3:** **We thank the reviewer for your insightful comments and agree that this work provides some insights into key drivers of HRQoL that may have implications for clinical management and policy interventions among people living with HIV in Peru, as well as Latin America & the Caribbean region.**

Specific Comments
Following a preliminary review of this article, I find this paper quite interesting and an important scientific contribution towards understanding how the effects of aging on HRQOL vary in individuals with or without HIV infections, and likewise in identifying the explanatory variables that account for these differences. However, there are specific issues that need to be addressed to improve the quality of this paper.

Abstract
Background: The aim of this study is not stated in the background, rather a feeble attempt was made to provide the purpose of this study in the result section. Therefore, these sections of the abstract must be revisited. It was not indicated why the study focused on women and not both sexes.
Method: The sampling technique used should be indicated and likewise the level at which alpha was set. It must be indicated whether the p-value was one-tailed or two-tailed to give proper context when interpreting it. The explanatory variables that were explored in the regression analysis must be mentioned.
Conclusion: The authors reported that “the physical component was significantly associated with physical activity, ethnicity, and chronic comorbidities while the mental component was significantly associated with physical activity, employment, and CD4+ cell count.” However, they did not mention the implication of these relationships in the conclusion. For instance, CD4+ count enables the assessment of HIV symptom severity which should have an impact on emotional wellbeing when such symptoms become more severe. Similarly, ethnicity alludes to socio-cultural factors which influence people's lifestyle and way of life. Therefore, the implications of these findings should be given the proper context to appreciate the implications of the findings of this study. It was stated in the conclusion that “Of note, physical activity affected both 47 components in the HIV-infected group, but with opposite associations,” for this reason, the results should at least state the variables with the most significant negative and positive values, and likewise the proportion of variance that accounted for the relationship or the effect size.

**Response to Reviewer #3: We appreciate the reviewer comments. In terms of the abstract: (1) we have updated the background so it is clearly stated that we are working with women with HIV (page 2, lines 28-30) and we continue to develop this idea on the main study. (2) the methodology has been updated to address the comments of the reviewer. (3) regarding the conclusion, we agreed with the reviewer’s recommendation to highlight the implications of these relationships and provide a holistic view of how to address quality of life. We have updated the conclusion on page 2 (lines 49 to 55).**

Main study
Background:
The background to this study is well written and provides a good rationale for the present study.

**Response to Reviewer #3: We appreciate the reviewer comments regarding the Background.**

Method:
1. The research design suggests that this is an observational cross-sectional study. It must be clearly stated so that there is a distinction bearing in mind that there are other types of cross-sectional study which fulfil other research purposes. Also, since this study was done among women with HIV and not AIDS, the defining cut-off point for the CD4+ cell count must be stated in the inclusion criteria.
2. It was not indicated how the sample size was determined to ensure that it has the power to detect the differences in the variables of interest between the study group and the comparison group.

**Response to Reviewer #3: Regarding the main study methodology, we have clarified that this is an observational cross-sectional study (page 5, line 111). We have added the cutoff point used for viral suppression on page 5 (lines 130-131) and the definition used for AIDS diagnosis on page 6 (line 141). Regarding our sample size, this study was a substudy of a larger study focused on vertebral fracture and sarcopenia outcomes therefore our overall sample size goals were somewhat larger than for this substudy. For this substudy, the primary outcomes of interest were physical and mental health component scores (PCS and MCS). Based upon prior literature from other countries comparing patients with and without HIV (with mean scores in range of 50, and standard deviation of 10) we estimated a sample of at least 396 patients (allocated in 2:1 ratio) would enable us to detect at least 6% difference in PCS or MCS scores between the two groups with 80% power at an alpha of 0.05. Of note, our final sample did not quite achieve the planned 2:1 ratio as we had to end recruitment somewhat earlier than anticipated due to the COVID-19 pandemic, however our final numbers per group still yielded 86% power for this substudy based upon our original parameters.**

Measures and data collection
In line 115, page 5, it was stated that a pilot study was conducted but the result of the pilot was not indicated as regards the validity and reliability of the test instrument. The validity and reliability of the test instruments - SF-36, IPAQ - must be indicated especially since it has been validated in Spanish.

**Response to Reviewer #3: In terms of the measures and data collection, we have updated *Measures of physical activity* and *SF36* sections on page 7 with more information regarding validity and reliability of both test instruments. The IPAQ has been validated in many languages including Spanish (line 151-154 page 6), same as the SF36 (lines 165-166, page 7), information about their validity has been updated on those lines.**

Data Analysis
The type of t-test statistic that was applied in the study should be stated – whether it was independent or the dependent t-test. It is important to indicate whether the p is one-tailed or two-tailed. This is important because this study is driven by a non-directional hypothesis in which case a two-tailed p-value should be appropriate.

**Response to Reviewer #3: In terms of data analysis, the descriptions of the t-tests and Mann-Whitney tests were updated to include that they were independent and two-tailed. (line 188, page 7).**

Results
1. The result section was well written.
2. However, the varied participants characteristics between the two groups show that they were not equivalent with respect to the variables that are likely to influence the HRQOL. First, the participants in the comparisons group were significantly older, more employed, of greater: BMI, adiposity, and white ethnic heritage. Given that some of these factors are also variate determinants of HRQOL, an explanatory model of the variance in the HQOL on the basis of other predictive variables may have errors except these covariates are appropriately controlled.
3. The results of the multivariate regression analysis should include the R-value to give a sense of the proportion of the population that accounted for the significant variance change in HQOL using the predictive model.

**Response to Reviewer #3: Thank you for these important comments. We recognize that there is variation in the participant characteristics mentioned between the HIV-infected and uninfected women, and therefore sought to adjust for these in our multivariable regression models. We agree with the suggestion to include the R^2^ values and now have added this to Table 4 (page 15).**

Discussion
1. The last sentence in paragraph one, page 19, reads as follows:
“As none of the aforementioned studies were conducted in Latin America nor specifically focused on women, a combination of changes in HIV treatment and socio-cultural nuances likely influenced the outcomes.”
2. I suggest that the sentence should be modified by replacing “a combination of changes” with “possible variations” since the authors are not certain of this claim.
3. I do not seem to agree with the interpretation of the findings highlighted in the last two sentences of the second paragraph on page 19 which stated as follows:
“However, for the HIV-infected subset, other factors such as mestizo ethnicity and presence of other comorbidities were independently associated with lower physical scores. These results suggest that despite progress made in the treatment for HIV, for aging Peruvian women, HIV continues to negatively impact the physical and sociodemographic aspects of their quality of life.”
In my view, the results indicated that those infected with HIV, and who were of mestizo ethnicity, were at a greater risk of poor physical functioning or physical health state than the rest. These hints of possible health disparity across ethnic boundaries that limit physical functioning in older women with HIV infection in Peru. However, given that such a relationship was not established among the control group, does not mean that it does not exist. Rather it could imply that HIV infection amplified some health disparities across socio-cultural boundaries in older women in Peru, in a manner that placed the mestizo ethnicity at a disadvantage. Since physical health in the HIV-infected population is also dependent on comorbidities, it implies that whatever factors that promote the onset of these comorbidities could be averse to physical health. Such factors may include limited access to healthcare, aging, prolonged usage of ART, socio-economic or employment (economic) inequality among others. Therefore, it could be investigated whether HIV-comorbidities are also common in the mestizo ethnicity than other ethnic groups and whether a similar trend was observed in studies done in other multi-ethnic countries in Latin America with similar access to health for all the ethnic groups as obtained in Peru.
4. The authors reported that physical activity has a mixed impact on the components of HRQOL since the emotional component was not improved in their study, and cited Goulding et al., 2019 (ref. 43). However, it would have been appropriate to also mention that the findings of Goulding et al., (2019) should be interpreted with caution because of the acknowledged limitations in the small sample size and large standard deviations recorded in the measured outcomes, which suggest that there was a wide margin of error or some degree of imprecision in the measurement. However, the authors had also mentioned that:
“In addition, multivariable regression adjusting for various factors including age, BMI, and other sociodemographic factors of the HIV-infected subset illustrated that physical activity levels are also correlated with both physical and mental components.”
Invariably, physical activity or exercise should be expected to influence physical and emotional HRQOL so far as the influence of age, BMI and socio-demographic factors are eliminated or controlled. Therefore, age, BMI and socio-demographic factors are confounding variables that may minimize the effects of an exercise intervention on HRQOL, and ought to be considered when interpreting the results of similar studies.

**Response to Reviewer #3: We greatly appreciate your comments on the Discussion section. We have replaced "a combination of changes" with "possible variations" (line 368, page 18). Regarding HIV and mestizo ethnicity and comorbidities, we agree with your thoughtful suggestions regarding the interpretation of these findings, and have updated those lines on page 18 (lines 377-380). We also have updated the sentence in reference to the Goulding *et al.* study acknowledging the limitations that this study presents so authors can interpret these results with caution (lines 417-419, page 20). We also agree that age, BMI and socio-demographic factors are confounding variables that may play impact the effects of an exercise intervention and should be considered when exploring the results of other similar studies.**

5. The authors in the introduction (see page 2, line 95), “hypothesized that HIV burden may be an important predictor for physical and mental health of this population.” However, it is not discussed whether this hypothesis was realized, and should be reflected in the discussion.

Conclusion
The conclusion section is meant to reflect on the outcome of the study concerning the set study objectives. Anything outside of this may distract from a primary need to project the key findings of the present study. I am wondering what the key objectives of this study are and whether they were met. I suggest that the specific objectives are clearly stated. Also, I do not find where a mention was made of whether the study hypothesis was accepted or rejected based on the findings.

**Response to Reviewer #3: Regarding the conclusion and hypothesis: as mentioned in the *Background*, we designed this study aiming to examine health-related quality of life, measured by the SF36, among women aging with and without HIV, as there is no previous data of this issue among women aging with HIV in Peru or other Latin-American countries. We hypothesized that HIV burden may play a role in terms of health-related quality of life, and our findings were consistent with this hypothesis. We have added this on lines 456-460 (page 21). We also emphasized directions for further studies in the last paragraph as we think it’s important to expand upon this work and further explore more determinants of health and health-related quality of life among women with HIV, which has been understudied in this region of the world.**

Orthographic check
There is a need for minor orthographic check of the manuscript.
Other Comments
This is a novel study which provided scientific findings that may have useful application in the clinical management and policy interventions of HIV disease.
Missing sections
The following sections are missing at the back end of this manuscript and should be provided by the authors in line with the journal format and style, including:
- List of Abbreviations
- Ethics approval and consent to participate
- Consent to publish
- Availability of data and materials
- Competing interests
- Funding
- Authors' Contributions

**Response to Reviewer #3: We have also double-checked the orthography of the manuscript. Furthermore, we have added a list of abbreviations, ethics approval, consent to participate, consent to publish and data availability at the end of the manuscript (page 26-27).**

This manuscript has not been previously published and is not under consideration in the same or substantially similar form in any other peer-reviewed media. It was previously presented as an abstract at the Consortium of Universities for Global Health 2021 Annual Conference. All authors listed have contributed sufficiently to the project to be included as authors. To the best of our knowledge, no conflict of interest, financial or otherwise, exists. We have included acknowledgements, disclosure statements and all funding sources with this submission.

Thank you again for your consideration.

Sincerely,

Evelyn Hsieh, MD, PhD

Section of Rheumatology, Allergy and Clinical Immunology

Yale School of Medicine

300 Cedar Street, TAC S-525 Po Box 208031 New Haven CT 06157

Email: [evelyn.hsieh@yale.edu](mailto:evelyn.hsieh@yale.edu)
