# Supplementary material for: Health-related quality of life among women aging with and without HIV in Peru
Source: PLoS One. 2022 Jun 9;17(6):e0269000. doi: 10.1371/journal.pone.0269000 (PMC9182248; doi:10.1371/journal.pone.0269000)
Supplement: Attachment — Submitted filename: Reviewer comment final.edited (1).docx [file pone.0269000.s001.docx]

**Review Report**

**Title:** Health-Related Quality of Life among Women Aging with and without HIV in Peru

**Reviewer: Prof. Sam Ibeneme**

**Institutional affiliations:**

1. University of Nigeria, Enugu Campus, Department of Medical Rehabilitation, Faculty of Health Sciences, Enugu, Nigeria. [sam.ibeneme@unn.edu.ng](mailto:sam.ibeneme@unn.edu.ng).
2. Department of Physiotherapy, Faculty of Health Sciences, School of Therapeutic Studies, University of the Witwatersrand, 7 York Road, Parktown, 2193 Johannesburg, South Africa

**Reviewers Report**

**General comments**

Many thanks for inviting me to review the suitability of the article *Health-Related Quality of Life among Women Aging with and without HIV in Peru’* for publication**.** Essentially, this article sought to (1) determine whether the effects of aging on HRQOL vary in aging women with or without HIV infections, and (2) to explore the explanatory variables for the variance in HRQOL in aging women with or without HIV infections using predictive models. Therefore, they compared older adult women with and without HIV infection to know whether the impact of aging on HRQOL is similar in both groups or whether there are differences in both groups which could be accounted for by HIV-related factors. The authors also explored the predictive models that could explain differences in HRQOL between the older adult women with or without HIV infection. The findings seem to demonstrate that HRQOL is more negatively affected among older adult women with HIV than otherwise. The summary score for the physical component of HRQOL in the HIV subset was significantly associated with physical activity, ethnicity, and chronic comorbidities suggesting a role for physical activity, socio-cultural factors and HIV-related/age-related complications in the determination of the physical wellbeing of the aging HIV-infected women. Similarly, the mental component was significantly associated with physical activity, employment, and CD_4_+ cell count suggesting a role for a physical activity lifestyle, social functioning/economic factors and symptoms of the disease severity in the determination of the emotional wellbeing of the aging HIV-infected individuals. Overall, it was shown that HRQOL may have important clinical application in the early identification of the key drivers of physical and emotional welling in the aging HIV infected individuals. These drivers should be prioritised in the clinical management and policy interventions of HIV disease which may vary across socio-cultural boundaries considering the impact of ethnicity. Thus, I consider this study to be original, useful, timely and relevant.

**Specific Comments**

Following a preliminary review of this article, I find this paper quite interesting and an important scientific contribution towards understanding how the effects of aging on HRQOL vary in individuals with or without HIV infections, and likewise in identifying the explanatory variables that account for these differences. However, there are specific issues that need to be addressed to improve the quality of this paper.

**Abstract**

**Background: T**he aim of this study is not stated in the background, rather a feeble attempt was made to provide the purpose of this study in the result section. Therefore, these sections of the abstract must be revisited. It was not indicated why the study focused on women and not both sexes.

**Method:** The sampling technique used should be indicated and likewise the level at which alpha was set. It must be indicated whether the p-value was one-tailed or two-tailed to give proper context when interpreting it. The explanatory variables that were explored in the regression analysis must be mentioned.

**Conclusion:** The authors reported that *“the physical component was significantly associated with physical activity, ethnicity, and chronic comorbidities while the mental component was significantly associated with physical activity, employment, and CD4+ cell count.”* However, they did not mention the implication of these relationships in the conclusion. For instance, CD_4_+ count enables the assessment of HIV symptom severity which should have an impact on emotional wellbeing when such symptoms become more severe. Similarly, ethnicity alludes to socio-cultural factors which influence people's lifestyle and way of life. Therefore, the implications of these findings should be given the proper context to appreciate the implications of the findings of this study. It was stated in the conclusion that *“Of note, physical activity affected both 47 components in the HIV-infected group, but with opposite associations,”* for this reason, the results should at least state the variables with the most significant negative and positive values, and likewise the proportion of variance that accounted for the relationship or the effect size.

**Main study**

**Background:**

The background to this study is well written and provides a good rationale for the present study.

**Method:**

1. The research design suggests that this is an observational cross-sectional study. It must be clearly stated so that there is a distinction bearing in mind that there are other types of cross-sectional study which fulfil other research purposes. Also, since this study was done among women with HIV and not AIDS, the defining cut-off point for the CD_4_+ cell count must be stated in the inclusion criteria.
2. It was not indicated how the sample size was determined to ensure that it has the power to detect the differences in the variables of interest between the study group and the comparison group.

**Measures and data collection**

In line 115, page 5, it was stated that a pilot study was conducted but the result of the pilot was not indicated as regards the validity and reliability of the test instrument. The validity and reliability of the test instruments - SF-36, IPAQ - must be indicated especially since it has been validated in Spanish.

**Data Analysis**

The type of t-test statistic that was applied in the study should be stated – whether it was independent or the dependent t-test. It is important to indicate whether the p is one-tailed or two-tailed. This is important because this study is driven by a non-directional hypothesis in which case a two-tailed p-value should be appropriate.

**Results**

1. The result section was well written.
2. However, the varied participants characteristics between the two groups show that they were not equivalent with respect to the variables that are likely to influence the HRQOL. First, the participants in the comparisons group were significantly older, more employed, of greater: BMI, adiposity, and white ethnic heritage. Given that some of these factors are also variate determinants of HRQOL, an explanatory model of the variance in the HQOL on the basis of other predictive variables may have errors except these covariates are appropriately controlled.
3. The results of the multivariate regression analysis should include the R-value to give a sense of the proportion of the population that accounted for the significant variance change in HQOL using the predictive model.

**Discussion**

1. The last sentence in paragraph one, page 19, reads as follows:

“*As none of the aforementioned studies were conducted in Latin America nor specifically focused on women,* ***a combination of changes*** *in HIV treatment and socio-cultural nuances likely influenced the outcomes.”*

2. I suggest that the sentence should be modified by replacing “***a combination of changes***” with “***possible variations***” since the authors are not certain of this claim.
3. I do not seem to agree with the interpretation of the findings highlighted in the last two sentences of the second paragraph on page 19 which stated as follows:

“*However, for the HIV-infected subset, other factors such as* *mestizo ethnicity and presence of other comorbidities were independently associated with lower physical scores. These results suggest that despite progress made in the treatment for HIV, for aging Peruvian women, HIV continues to negatively impact the physical and sociodemographic aspects of their quality of life*.”

In my view, the results indicated that those infected with HIV, and who were of mestizo ethnicity, were at a greater risk of poor physical functioning or physical health state than the rest. These hints of possible health disparity across ethnic boundaries that limit physical functioning in older women with HIV infection in Peru. However, given that such a relationship was not established among the control group, does not mean that it does not exist. Rather it could imply that HIV infection amplified some health disparities across socio-cultural boundaries in older women in Peru, in a manner that placed the mestizo ethnicity at a disadvantage. Since physical health in the HIV-infected population is also dependent on comorbidities, it implies that whatever factors that promote the onset of these comorbidities could be averse to physical health. Such factors may include limited access to healthcare, aging, prolonged usage of ART, socio-economic or employment (economic) inequality among others. Therefore, it could be investigated whether HIV-comorbidities are also common in the mestizo ethnicity than other ethnic groups and whether a similar trend was observed in studies done in other multi-ethnic countries in Latin America with similar access to health for all the ethnic groups as obtained in Peru.

4. The authors reported that physical activity has a mixed impact on the components of HRQOL since the emotional component was not improved in their study, and cited Goulding *et al.,* 2019 (ref. 43). However, it would have been appropriate to also mention that the findings of Goulding *et al., (*2019) should be interpreted with caution because of the acknowledged limitations in the small sample size and large standard deviations recorded in the measured outcomes, which suggest that there was a wide margin of error or some degree of imprecision in the measurement. However, the authors had also mentioned that:

*“In addition, multivariable regression adjusting for various factors including age, BMI, and other sociodemographic factors of the HIV-infected subset illustrated that physical activity levels are also correlated with both physical and mental components.”*

Invariably, physical activity or exercise should be expected to influence physical and emotional HRQOL so far as the influence of age, BMI and socio-demographic factors are eliminated or controlled. Therefore, age, BMI and socio-demographic factors are confounding variables that may minimise the effects of an exercise intervention on HRQOL, and ought to be considered when interpreting the results of similar studies.

5. The authors in the introduction (see page 2, line 95), *“hypothesized that HIV burden may be an important predictor for physical and mental health of this population*.” However, it is not discussed whether this hypothesis was realised, and should be reflected in the discussion.

**Conclusion**

The conclusion section is meant to reflect on the outcome of the study concerning the set study objectives. Anything outside of this may distract from a primary need to project the key findings of the present study. I am wondering what the key objectives of this study are and whether they were met. I suggest that the specific objectives are clearly stated. Also, I do not find where a mention was made of whether the study hypothesis was accepted or rejected based on the findings.

**Orthographic check**

There is a need for minor orthographic check of the manuscript.

**Other Comments**

This is a novel study which provided scientific findings that may have useful application in the clinical management and policy interventions of HIV disease.

**Missing sections**

The following sections are missing at the back end of this manuscript and should be provided by the authors in line with the journal format and style, including:

- List of Abbreviations
- Ethics approval and consent to participate
- Consent to publish
- Availability of data and materials
- Competing interests
- Funding
- Authors' Contributions
