# Supplementary material for: Health-related quality of life among women aging with and without HIV in Peru
Source: PLoS One. 2022 Jun 9;17(6):e0269000. doi: 10.1371/journal.pone.0269000 (PMC9182248; doi:10.1371/journal.pone.0269000)
Supplement: Attachment — Submitted filename: Reviewer comment final.edited (1)- revision 1.docx [file pone.0269000.s003.docx]

PLOS ONE

PONE-D-21-11937R1

**Review Report**

**Title:** Health-Related Quality of Life among Women Aging with and without HIV in Peru

**Reviewer: Prof. Sam Ibeneme**

**Institutional affiliations:**

1. University of Nigeria, Enugu Campus, Department of Medical Rehabilitation, Faculty of Health Sciences, Enugu, Nigeria. [sam.ibeneme@unn.edu.ng](mailto:sam.ibeneme@unn.edu.ng).
2. Department of Physiotherapy, Faculty of Health Sciences, School of Therapeutic Studies, University of the Witwatersrand, 7 York Road, Parktown, 2193 Johannesburg, South Africa

**Reviewers Report**

**General comments**

Many thanks for inviting me to review the revised article *Health-Related Quality of Life among Women Aging with and without HIV in Peru’* for publication**.**

**Specific Comments**

I have gone through the revised manuscript and feel satisfied that the authors reflected all the corrections as required. The quality of this manuscript has greatly improved and is fit for publication.

**Recommendation**

I recommend that this article is accepted for publication as it is.
